# Development and modelling of realistic retrofitted Nature-based Solution scenarios to reduce flood occurrence at the catchment scale

**DOI:** 10.1007/s13280-020-01493-8

**Published:** 2021-01-26

**Authors:** Valerie Chen, Jose Ricardo Bonilla Brenes, Fernando Chapa, Jochen Hack

**Affiliations:** 1 Department of Civil and Environmental Engineering, Technical University Darmstadt, Franziska-Braun-Str. 7, 64287 Darmstadt, Germany; 2 Research Group SEE-URBAN-WATER, Section of Ecological Engineering, Institute of Applied Geosciences, Technical University Darmstadt, Schnittspahnstr. 9, 64287 Darmstadt, Germany

**Keywords:** Green urban rivers, Infrastructure, LID, Nature-based solutions, Stormwater management, Upscaling

## Abstract

Decentralized Nature-based Solutions such as Urban Green Infrastructures (UGI) are increasingly promoted to reduce flooding in urban areas. Many studies have shown the effectiveness of flood control of UGI at a plot or neighbourhood level. Modelling approaches that extrapolate their flood reducing impact to larger catchment scales are often based on a simplistic assumption of different percentages of UGI implementation. Additionally, such approaches typically do not consider the suitable space for UGI and potential implementation constraints. This study proposes a scenario development and modelling approach for a more realistic upscaling of UGI based on empirical insights from a representative neighbourhood. The results from this study, conducted in the metropolitan area of Costa Rica, show that upscaling the full potential for UGI could significantly reduce surface runoff, peak flows, and flood volumes. In particular, the permeable pavement has the highest potential for flood reducing in public space while cisterns perform best at the property level. These results can guide the formation of policies that promote UGI.

**Supplementary Information:**

The online version of this article (10.1007/s13280-020-01493-8) contains supplementary material, which is available to authorized users.

## Introduction

There is a clear trend towards urbanisation in the world. Today, 50% of the world’s population lives in urban areas and this number is expected to rise by 2050 (United Nations 2015). Urbanisation impacts the urban hydrology by altering the water balance of cities (Barbosa et al. 2012; Walsh et al. 2012; Marchionni et al. 2019). Impermeable surfaces and hydraulically efficient drainage networks decrease the infiltration and evapotranspiration volume, increasing the amount of superficial runoff (Walsh et al. 2005; Brown et al. 2009). In consequence, extreme precipitation events increase the likelihood of flooding.

Nature-based Solutions (NbS) such as Urban Green Infrastructures (UGI) are being promoted (European Commission 2015) to deal with socio-ecological issues caused by increased urbanisation. In contrast to grey infrastructure, NbS rely on near-natural structures and processes to reduce stormwater runoff and improve water quality. Examples of UGI are retention basins, roof greening, infiltration trenches, and permeable road surfaces (Barbosa et al. 2012; Li et al. 2019a, b).

UGI solutions are increasingly implemented at small scales (i.e. street level) around the world (Jia et al. 2014, 2015; Joksimovic and Alam 2014; Sin et al. 2014; Baek et al. 2015; Palla and Gnecco 2015; Versini et al. 2015; Chui et al. 2016; Li et al. 2019a, b). Hydrological models, including simulation of UGI under different meteorological conditions can support policy-making for infrastructure development. By modelling and comparing hypothetical scenarios, new insights regarding the hydrological response (e.g. occurrence, magnitude, timing, and duration of flooding) can be revealed (Golden and Hoghooghi 2018). The closer the model representation to the actual infrastructure scenario is, the more ‘representative the modelling outputs are.

In urban contexts, the development of UGI scenarios must consider existing spatial constraints (e.g. limited availability of space, existing uses of space for traffic or housing, regulations regarding the placement as well as technical design criteria of UGI) to be feasibly implemented. In in a review of studies regarding catchment-wide upscaling of UGI by Golden and Hoghooghi (2018), the authors argued that a key consideration for upscaling and applying UGI models is a meaningful placement of those UGI practices at the catchment scale. Contrary to conventional urban drainage infrastructure, retrofitted UGI is embedded as permeable and/or vegetated areas within an urban matrix and occupy space not only sub-superficially but also on the surface potentially requiring alternative use of space. Therefore, the model representation should be as realistic as possible, reflecting the actual potential for different UGI development strategies.

However, studies do not account for site-specific constraints, and instead focus more on potential performances of UGI at different degrees of implementation. Palla and Gnecco (2015), for instance, modelled scenarios based on land use change related to UGI (e.g. different percentages of rooftops converted to green roofs, and 16% of road and parking lots converted into permeable pavement), but without considering the suitability of the sites. Similarly, Joksimovic and Alam (2014) assumed scenarios for six types of UGI based on Land Use/Land Cover (LULC) characteristics, but also did not account for the number of available spaces for implementation. Site-specific aspects related to UGI were also omitted in studies relating to LULC conversion (Chaosakul et al. 2013; Jia et al. 2014), cost and runoff reduction potential (Jia et al. 2015), first flush contamination (Baek et al. 2015), or budget constraints and overall area conversion limits (Liang et al. 2019).

In this study, we argue that to enable more effective policy-making and promotion of UGI, there is a need for data about site-specific constraints for the implementation of UGI, which can provide more insights than arbitrarily considering different degrees of fictive UGI implementation. In the context of retrofitting UGI, considering physical, regulatory and social constraints to the implementation of UGI is especially relevant (Neumann and Hack 2019).

The scenario developing approach adopted in this study address this need. To capture those existing constraints in a given landscape configuration, this study proposes to use detailed information from a representative area, in this case a residential neighbourhood, and spatially upscale this information to other residential areas within the catchment. The area was chosen because it is located within a relevant runoff generating part of the urban catchment where retrofitted UGI implementation could contribute to reducing flood occurrence downstream.

This study presents the simulation of UGI scenarios in the hydrological model PC Storm Water Management Model (PCSWMM; Rossman and Huber 2016). The study includes data about the availability of space and realistic constraints such as use and social acceptance of UGI in public space, and traffic limitations for UGI implementation retrieved from field studies conducted in 2019 in the same neighbourhood (Towsif Khan et al. 2020; Rose 2020; Fluhrer et al. 2021). Based on the representativeness of the site, a more realistic UGI scenario is simulated considering a catchment-wide implementation. The objectives of this study are (a) to build realistic UGI development model representations, and (b) to estimate the potential relative and combined contributions of retrofitted UGI implementation in public areas.

This UGI scenario approach is tested and discussed in the context of a highly urbanized tropical catchment, located in the Great Metropolitan Area of Costa Rica. Rapidly increasing urbanization in the area has caused a series of flooding events that have damaged public and private infrastructure (Chaves Herrera et al. 2014; Oreamuno and Villalobos 2015). As a consequence, the Constitutional Court ordered municipalities and other relevant institutions to solve the water management issues in an integrated manner. The authors believe that this study can guide decision-makers on possible remediation strategies considering UGI at a catchment scale.

## Materials and methods

### Study area

The Quebrada Seca–Burío River flows through the north-western part of the Great Metropolitan Area of Costa Rica, crossing the municipalities of San Rafael, Barva, Heredia, Flores, and Belén. About 63% of its 23 km^2^ catchment area has been urbanized (Oreamuno and Villalobos 2015). The average annual precipitation is 2042 mm with an average temperature of 24.8 °C (Chaves Herrera et al. 2014) and an altitude gradient between 869 and 1626 m.a.s.l. The catchment is located on the Pacific side of the country, being characterized by a well-defined rainy season that lasts from May to October.

The river has a sinuous morphology, having increased both in-depth and width due to increasing hydraulic stress caused by the discharge of high volumes of urban runoff.

Most of the streets are paved, following rectangular block dead-end network patterns. Stormwater runoff is discharged directly into the river by sewer drainage systems. Most of the residential houses have a septic tank that stores and infiltrates black water. Due to the reduced volume of septic tanks and avoidance to pay private companies emptying them, grey water is usually discharged along the streets. Although the riparian zone is protected within 10 m parallel to the stream, many buildings and streets have been built right next to the river.

### Model setup

The software PCSWMM Version 7.2.2785 (Rossman and Huber 2016) was employed to simulate the rainfall–runoff generation. It is a dynamic model that combines the US EPA Storm Water Management Model (SWMM) with a Geographic Information System (GIS). The hydraulic transport component included in the software guides the runoff resulting from a sub-catchment division through a system of pipes, channels, storage and treatment devices, pumps, and regulators. In a simulation period consisting of several time steps, ranging from seconds to days, for each sub-catchment the resulting runoff and for each conduit (pipe or channel), flow rate and flow depth, are modelled. In PCSWMM several hydrological and hydraulic processes can be modelled such as time-varying rainfall, evaporation of standing surface water, rainfall interception from depression storage, flow routing through conduit networks, surcharging, and surface ponding. Various types of UGI practices can also be modelled to capture and retain rainfall-runoff, namely bio-retention cells, rain gardens, green roofs, infiltration trenches, continuous permeable pavement, rain barrels/cisterns, rooftop disconnections, and vegetative swales (Rossman and Huber 2016).

#### Model input data

Input data requires information on the characteristics of the catchment. These include the size of the area, average width and slope of each sub-catchment, precipitation data, the fraction of impervious area, weighted roughness coefficients of the previous and impervious area according to Manning (1891) for the overland flow (SWMM uses the Manning equation to calculate the flow rate), and the depression storage for permeable and impermeable areas (Rossman 2015).

**Fig. 1:**
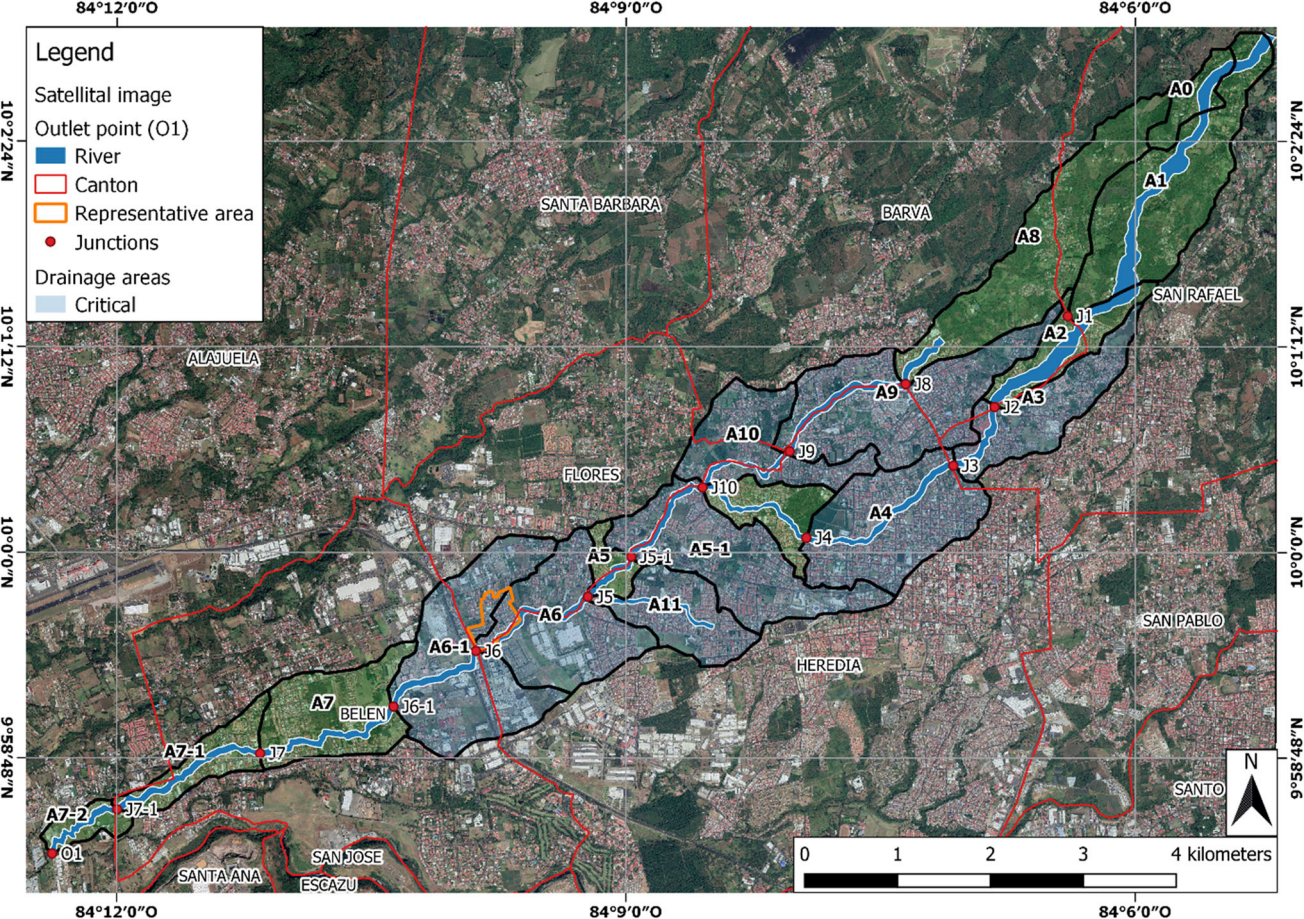
Map of the model structure showing the 17 sub-catchments (Ai; 8 blue-shaded considered as critical for flood generation), 13 river junctions (Ji), the representative area (in orange) used to estimate the UGI potential and the outlet (O1) of the catchment. *Source of background image* Google Earth

Based on the approaches of Gironás et al. (2009), Ji and Qiuwen (2015), and Lhomme et al. (2004), the digital elevation model (DEM) obtained from Instituto Geográfico Nacional (2019) with a 5 × 5 m pixel resolution, was manually processed to delineate the sub-catchments. They were defined based on the natural hydrology (using the DEM) and the geometry of the drainage system, since urban catchments represent a mixture of natural terrain and artificial drainage. The channels of the drainage system were assumed along with the street network. The street network within the study area was downloaded from “OpenStreetMap” as OSM format and converted into a line shape file. In the DEM, the elevation of the cells along the street network was lowered by 5 m. Similarly, the elevation of buildings was increased to consider them as obstacles to the floodway during their representation in the model. After DEM processing, the 17 sub-catchments were delineated in QGIS 3.6.0 considering anthropogenic changes to floodways (i.e. streets and other urban features) and critical sites of the river (e.g. bridges). Figure 1 shows those 17 sub-catchments, 13 river-junctions and the outlet of the catchment, as well as the representative area (indicated in orange) which was used to estimate the UGI implementation potential in urbanized sub-catchments. The annotations of the sub-catchments Ai and junctions Ji derive from the development of the model.

**Fig. 2:**
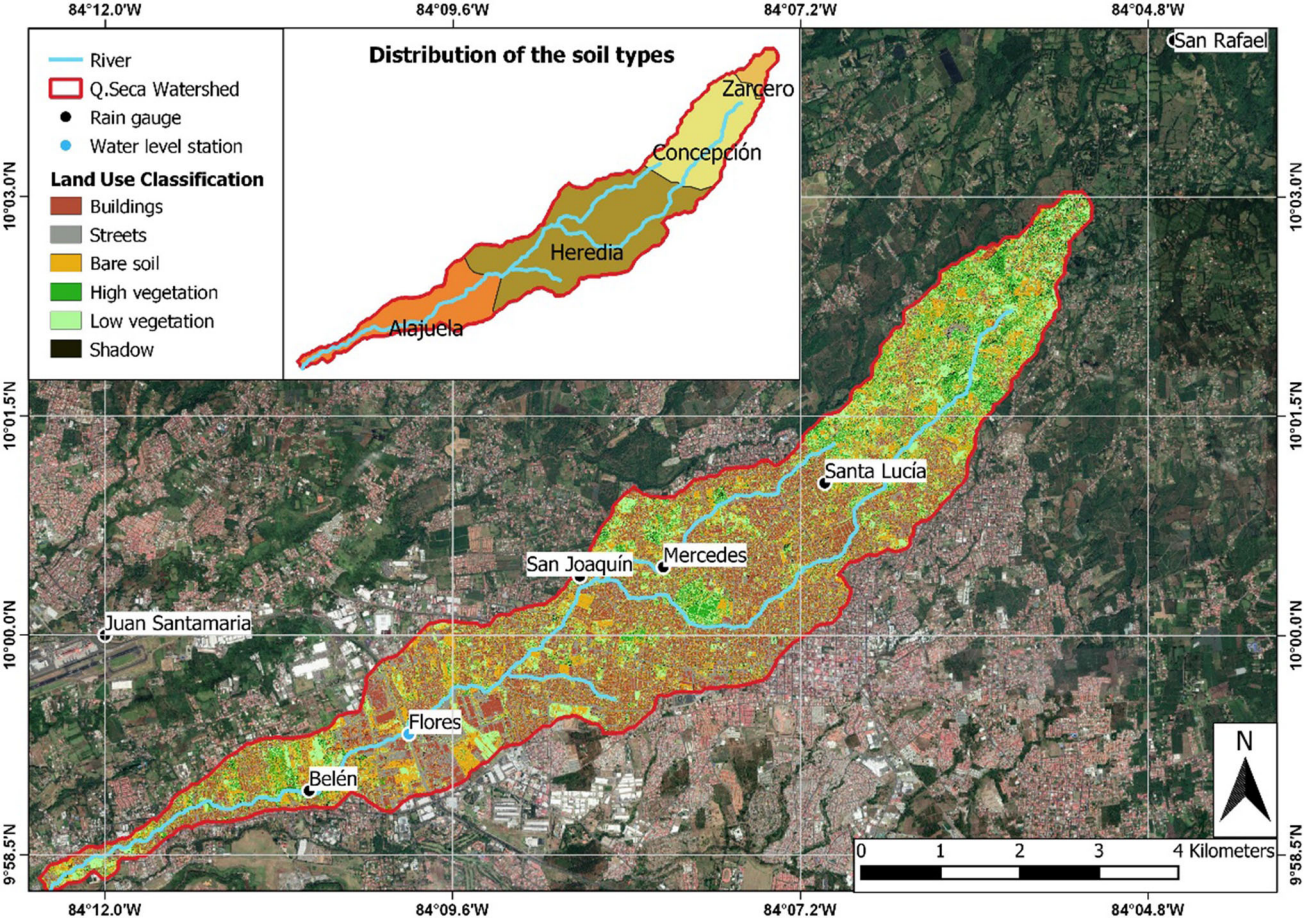
Land-use classification, location of the rain gauges and water level station, and distribution of the soil types in the catchment. *Source of background image* Google Earth

The LULC classification was based on a true colour image methodology (Chapa et al. 2019), using the Semi-Automatic Classification Plugin 6.2.9 in QGIS 3.6.0. A satellite image was exported from Google Earth Pro 7.3.2, with a pixel resolution of 0.5 m. Based on “Regions of Interest” representing polygons of homogenous areas (Congedo 2019), the LULC was classified according to the following categories: buildings, streets, bare soil, high vegetation, low vegetation, and shadows. Shadows were considered to avoid misclassification of certain areas, especially nearby buildings and trees. Areas classified as shadow corresponds to about 2% of the total area. Water bodies were also classified as shadows due to the similarity of their image colour, influencing a percentage lower than 1% of the classification. Figure 2 shows the LULC classification. Additionally, soil data was obtained from the soil database of the Ministry of Agriculture and Livestock of Costa Rica (Oreamuno and Villalobos 2015). The soil distribution consists of loam, loam/silt, fine clay, and silt/clay, named as Zarcero, Concepción, Heredia and Alajuela in Fig. 2.

Table S1 in shows the characteristics of the 17 sub-catchments of the Quebrada Seca considered in the PCSWMM model. Eight sub-catchments, highlighted in grey in Table S1, are considered as critical due to their high degree of impervious area and relative share of contribution to flooding. These sub-catchments are assumed to be similar to the representative area. UGI scenarios are modelled only for these sub-catchments.

Monthly mean values of pan-evaporation measurements were retrieved from the website “UNdata” (United Nations 2010) for the Station Juan Santamaría located about 2 km outside of the catchment. The mean values correspond to potential evaporation and were converted to actual evaporation using a factor of 0.7 accordingly to the SWMM User’s Manual (Rossman 2015).

The model performance of UGIs were analysed on the basis of three hydrological conditions. The first condition (event 1) considered 4 months of rainfall data corresponding to the rainy season and registered by four stations located within the basin, the second and third conditions were defined by two extreme precipitation events associated with storms within a 10 year (event 2) and 50 years (event 3) period. Runoff data series used for event 1 corresponded to precipitation volumes recorded every 5 min, covering a period from July to October 2019 which represents the rainy season. Runoff data was obtained from water level measurements in Flores (see Fig. 2). A hydrostatic pressure sensor was located under a bridge to record elevation measures allowing to calculate flow discharge based on the Gauckler–Manning formula (Gauckler 1867; Manning 1891).

Precipitation data from four stations (see Fig. 2) with a temporal resolution of 5 min available since July 2017 were provided by a local municipality. Due to the limitation of the runoff data, which was only available since the end of June 2019, rainfall data was also used from the same 4-month period. Events 2 and 3 were described in a previous study (Oreamuno and Villalobos 2015). The generation of extreme events implied the analysis of the spatial and temporal distribution of punctual precipitation events that generated floods from 2001 to 2014. Hourly precipitation data were used to model each storm event. The results were combined with estimated precipitation volumes collected by the Juan Santamaría Airport and Santa Lucía Meteorological Stations. This enables the generation of synthetic events both for the 10 and 50-year return period.

Table S2 summarizes the model input data and provides information about its resolution, source, date of origin/period of time and processing.

#### Model calibration and validation

To calibrate the model, a sensitivity analysis of the hydrological response of the catchment scale was carried out using the Sensibility based Ratio Tuning Calibration (STRC) tool provided by PCSWMM. All modifiable parameters were analysed to identify their level of uncertainty in the calculations. They were identified based on the STRC results and compared with previous results that have shown a relatively high degree of uncertainty (Choi and Ball 2002). Parameters analysed were catchment width, impervious percentage area, curve number, storage depth on impervious area, storage depth on pervious area, drying time, zero impermeability percentage area, and percentage routed. The curve number (USDA 1986) and catchment width were identified as the most sensitive parameters. For more detailed information about infiltration parameters refer to Rossman and Huber (2016). Subsequently, the model was calibrated by varying these parameters within physically plausible limits for 2 months (July–August) of the rainfall–runoff data. Nash–Sutcliffe-efficiencies (NSE) of 0.503 and a mean square error (*R*^2^) of 0.636 were achieved.

The model was validated using the rainfall–runoff data of the months of September–October, considering the same model parameterization. Error coefficients for the validation period were satisfactory, with an NSE of 0.758 and a *R*^2^ of 0.774. Since the hydrological data were grouped into two periods, the model was developed with the data set for calibration. The validation data were used to estimate whether the model works with similar characteristics using independent registers. The NSE is a parameter used to estimate the simulation adjustment level. Both cases have similar performances, which is the expected result. The fact that the validation data fit the model better shows that the model is reliable. The results of the model’s calibration and validation are summarized in Appendix S3.

Given these results, it is assumed that the model is set up sufficiently well to simulate the rainfall–runoff characteristics of the catchment and that it can be reliable used for the intended scenario modelling of UGI. The final model parameterization is summarized in Tables S3 and S4.

### Urban Green Infrastructure scenarios

To model the potential impact of UGI on flooding, two distinct scenarios of combinations of UGI options were developed: UGI scenario 1 (S1) is based on an detailed fieldwork assessment of the maximum spatial potential to implement UGI within the available public space (Fluhrer et al. 2021), and UGI scenario 2 (S2) is a complementary scenario for UGI implementation on private properties. Additionally, each UGI element (permeable pavement, bio-retention cell, infiltration trench, detention basin, cistern, green roof) included in each of the two scenarios is modelled separately to assess their individual contributions. Modelling is carried out for the three rainfall events to assess the UGI performances under different hydrological conditions.

The principal aim of this methodological work is to define meaningful and realistic scenarios of retrofitted UGI for urban areas of the catchment-based on detailed empirical observations from a representative residential neighbourhood within the catchment. The representative neighbourhood is a closed urban drainage area located in the middle of the watershed (Fig. 1). It was selected as an experimental site of the SEE-URBAN-WATER Research Project based on a participatory process where different Costa Rican municipalities proposed experimental sites to study the implementation of UGI at the neighbourhood scale, and it has been studied and monitored in detail for over 10 months since March 2019 (Neumann and Hack 2019; Towsif Khan et al. 2020; Rose 2020; Fluhrer et al. 2021). To assure the representativeness of the area, several considerations were taken into account: (1) The area makes up 2.4% of the catchment area, (2) onsite visits confirmed similar spatial characteristics at the site and the entire watershed, such as the width of streets, sidewalks and green verges, use of public space, building structures, distribution in space of roads of different hierarchy and unbuilt areas, (3) based on the analysis of remote sensing images, the history of urbanization process patterns occurred similarly in time and space, (4) the LULC distribution are similar at the site and watershed scale. With these considerations, the authors believe that the representativeness can be assumed to a sufficient degree for the given modelling purpose. Based on these empirical observations, the following scenario (S1) is defined in terms of their constraints and potential for implementation. This scenario is meant to be realistic in terms of its technical feasibility of implementation regarding space availability and typical constraints of urban areas as well as meaningful regarding its socio-political promotion of upscaling.

The maximum potential for retrofitted UGI in public spaces (streets, sidewalks, unbuilt open spaces) to reduce surface runoff peaks and volume was investigated in the experimental site (Fluhrer et al. 2021). The UGI scenario S1 resulting from this investigation considers four UGI options to be modelled with PCSWMM: bio-retention cells, infiltration trenches, permeable pavement and detention basins. To identify potential sites for these UGI options, available green and unbuilt space along streets, sidewalks, parcels and the riverfront were evaluated. The potential of sites was limited by taking into account physical constraints due to individual street designs (width of the street, gutter, green verges and sidewalks), road types based on the level of traffic intensity and road hierarchy (Fig. S1), and car entries to properties. Property ownership for public properties and land-use and areas considered as suitable for the placement of UGI within the representative neighbourhood are illustrated in Fig. S2.

Additionally, the following constraints to UGI implementation that cannot be illustrated in figures, were also considered: car entries to private properties, space requirements defined in technical guidelines for the different UGI elements, regulations regarding the placement of street greenery in public space, and resident preferences for the placement of UGI. To measure the latter constraints, residents from the area were interviewed with regard to their opinion on using existing green verges for water treatment, aesthetic upgrading of green verges with plants, and the occupancy of street space for additional green spaces (Rose 2020). While the acceptance for the use of existing green verges for water treatment and aesthetic upgrading of green verges with plants was high, the use of street space for additional UGIs was rejected by almost two thirds of the interviewed residents (*N* = 154). Consequently, only moderate UGIs interventions at favourable sites in the street network, such as bio-retention cells at street corners, and to a higher degree UGI options to be implemented without changing the current functionality of spaces (e.g. infiltration trenches along green verges) were considered (Fluhrer et al. 2021).

Based on these assessments, the potential space for implementation of each UGI option was determined (Fig. S7) and translated, given the land-use classification of the neighbourhood, to representative percentages of land-uses suitable for conversion into the four UGI options (see Table S2). These percentages are the maximum potential for the four UGI options for a given land-use distribution as retrofitted measures in public space assuming that all suitable and available space for UGI would be used. Maps showing the potential sites for all four UGI options for the experimental neighbourhood are included in Figs. S3, S4, S5 and S6.

These representative values are then upscaled to other urbanized sub-catchments represented in the model used for this study. Eight of the seventeen sub-catchments were considered for the UGI implementation simulation. They cover 47% of the entire catchment and are considered as critical areas for flood generation since they produce around 77% of the total runoff volume in the baseline scenario. The upscaling was done by applying a linear relationship between the representative UGI percentages for the four UGI options from the experimental site to the land-use distribution of the eight sub-catchments. Hence, an assumption is that areas with similar LULC have a similar potential for implementation of UGI. The latter because urbanization of most of the catchment has occurred at the same time and in a similar manner under the same urban planning regulations (Weyand 2020). Since this UGI implementation scenario (S1) only considers public space, the principal agents to promote this transformation are public authorities.

UGI scenario S2 was developed to consider green roofs as UGI options on private properties. This scenario is not intended to represent a realistic scenario to the same degree as S1, because the technical suitability of roofs and social acceptance were not analysed. The scenario considers that 25% of buildings converted into green roofs. Thus, 25% of the area classified as buildings was divided into 100 m^2^ units representing the roof area of houses. Similarly, 75% of areas defined as buildings are assumed to install rain barrels of 10 m^3^ volume. Here, 75% of the area classified as buildings was divided in 150 m^2^ properties. These values represent typical property size identified onsite (Fluhrer et al. 2021). Finally, all UGI elements were modelled individually in order to compare individual performances. Table 1 summarizes the assumptions regarding land-use conversions into UGIs for the two considered scenarios.

**Table 1:** Representative percentages of land-uses suitable for conversion into the four UGI options

UGI element	Scenario 1 (S1)		Scenario 2 (S2)	
	Percentage conversion (%)	Land-use class converted	Percentage conversion (%)	Land-use class converted
Permeable pavement	17.5	Streets		
Bio-retention cell	6.5	Streets		
Infiltration trench	3.0	Streets		
Detention basin	0.015	Bare soil, low vegetation		
Cistern			75	Buildings
Green roof			25	Buildings

Event 1 was modelled based on precipitation measurements of the rainy season of 2019 (July–October) for the evaluation of a 4-month performance with reoccurring rainfall. And two single flood-causing events—synthetic rainfall events with statistical return periods of 10 years (event 2) and 50 years (event 3)—obtained from Oreamuno and Villalobos (2015) based on past precipitation events with flooding impacts (Table 2).

**Table 2:** Specifications of precipitation events used to model the performance of UGI scenarios

Event	Duration	Return period (years)	Total volume (mm)	Maximum precipitation intensity for each meteorological station (mm/h)						
				San Jose	Juan Santamaría	Santa Lucía	San Rafael	Mercedes	San Joaquin	Belen
1	July–October 2019	–	980.2				85.3	128.0	167.6	106.1
2	330 min	10	67.6	85.7	109.2	166.5				
3	330 min	50	110.4	140.0	178.3	271.9				

Runoff volume, runoff peak, and runoff coefficient at either the junctions of the drainage network or the outlets of sub-catchments indicated in Fig. 1 are the principal model outputs used for the assessment and comparison of the different UGI scenarios and the status quo (current land-use and drainage conditions). Additionally, model outputs at critical points of the river network (junctions in the model structure) were analysed to assess the performance of UGI scenarios at most critical sites within the catchment.

## Results

### Modelling results of UGI scenarios for each rainfall event

For all the rainfall events, the results show that both UGI scenarios S1 (streets and open spaces) and S2 (properties) reduce surface runoff generation compared to the current situation, but in different magnitudes (Table 3). S1 results, on average, in a higher reduction of runoff generation compared to S2 (measures at properties). The total runoff volume in all rainfall events is reduced by at least 50% in S1 whereas in S2 it is at most a reduction of 12.4% (10-year rainfall event). The gap in performances is similar for the reduction of the runoff coefficient, but shorter for the reduction of peak runoffs. Peak runoff is reduced in S1 by at least 55% and to a maximum of 33.3% in S2.

**Table 3:** Percentages of reductions compared to the status quo modelling results total runoff volume, average peak runoff, and runoff coefficient for both UGI scenarios and different rainfall events

Rainfall event	UGI scenario	Runoff volume (%)	Peak runoff (%)	Runoff coefficient (%)
1 (4-month duration)	S1	60.3	55.7	60.5
	S2	8.0	21.5	8.0
2 (10-year event)	S1	57.2	57.9	58.5
	S2	12.4	33.3	13.8
3 (50-year event)	S1	50.5	58.4	51.0
	S2	8.4	14.2	8.7

Some general trends can be identified in the performance of both scenarios concerning the two event-based rainfall events (50- and 10-year). In both cases, the percentage of total runoff volume reduction and the runoff coefficient decreases with a higher amount and intensity of rainfall. However, for S1, the percentage of peak runoff reduction still increases with higher rainfall amounts and intensities while for S2 it significantly decreases (from 33.3 to 14.2%).

**Fig. 3:**
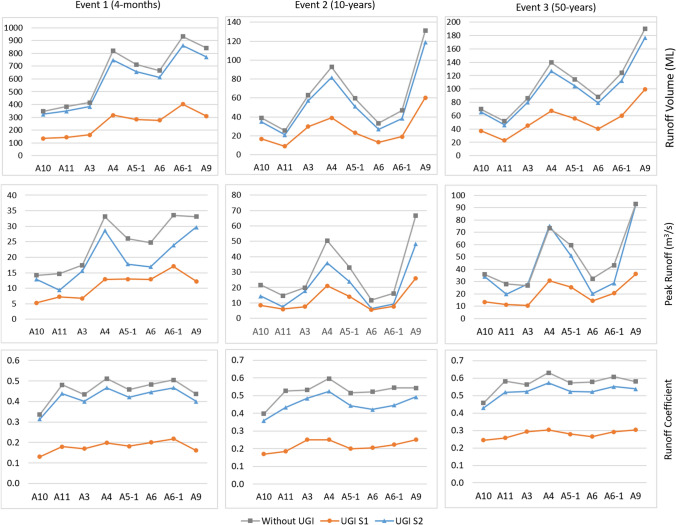
Results for runoff volume, peak runoff and runoff coefficient of UGI scenarios compared to modelling results without UGI implementation for the three considered rainfall events

The spatially distributed performance of S1 and S2 compared to the status quo for the three rainfall events is visualised in Fig. 3. S1 shows in all cases there are higher reductions for total runoff volume, peak runoff, and runoff coefficient in comparison to S2.

**Fig. 4:**
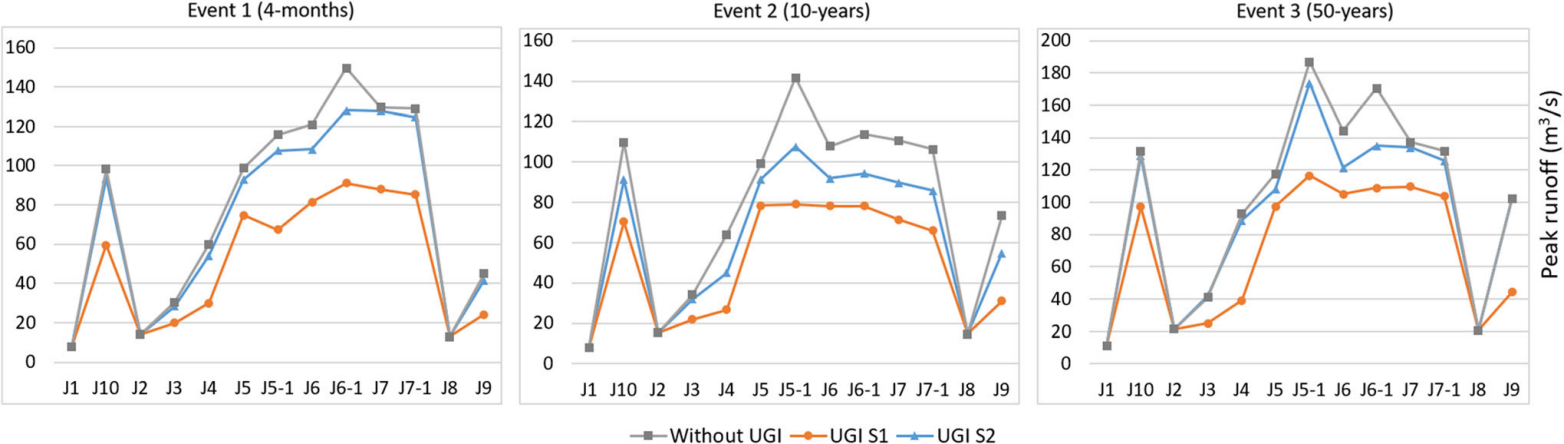
Modelling results for peak runoff at junctions of UGI scenarios compared to modelling results without UGI implementation for the three considered rainfall events

It is important to reduce peak runoff at different points along the river course to reduce river bed and bank erosion, reduce hydraulic stress to the river ecosystem, and aid the protection of key structures (e.g. bridges or bank stabilizations). Figure 4 illustrates how peak runoff is decreased at different junctions within the catchment by the different UGI scenarios. Compared to the peak runoff reduction at the outlet of each sub-catchment (see Fig. 3), the peak runoff at different junctions of the catchment reflects also the specific structure and topology of the river network (Fig. 4). Again, S1 achieves significantly higher reductions than S2. The reductions of peak runoff for the S1 for all rainfall events vary between 17% and almost 60% whereas the reductions of the S2 vary between near 0% to almost 30% (Fig. 4).

The peak runoff and total runoff volume reducing effect of UGI scenarios at the catchments’ outlet can be considered as an integral indicator for the catchment-wide effectiveness and is of concern for downstream parties. Table 4 summarizes these results for all rainfall events. S1 results in avoidance of several flood occurrences within the catchment, much higher reduction in peak runoffs, and reduction of total runoff volumes than S2.

**Table 4:** Peak runoff and total runoff volume at the catchment outlet (O1) of UGI scenarios compared to modelling results without UGI implementation for the three considered rainfall events

Rainfall event	Peak runoff at catchment outlet (m^3^/s)								Total runoff volume at catchment outlet (ML)					
	S1				S2				S1			S2		
	Without UGI	With UGI	%	No. of junctions flooding avoided	Without UGI	With UGI	%	No. of junctions flooding avoided	Without UGI	With UGI	%	Without UGI	With UGI	%
1 (4-month)	114.4	84.1	26.5	6	114.406	114.4	0.0	0	8020	5030	37.3	8020	7650	4.6
2 (10-year)	99.3	59.6	40.0	2	99.3	79.9	19.6	1	2010	1820	9.5	2010	2020	− 0.5
3 (50-year)	113.7	97.8	14.0	4	113.7	112.9	0.7	0	2320	2160	6.9	2320	2400	− 3.4

### Modelling results for each UGI element for each rainfall event

**Fig. 5:**
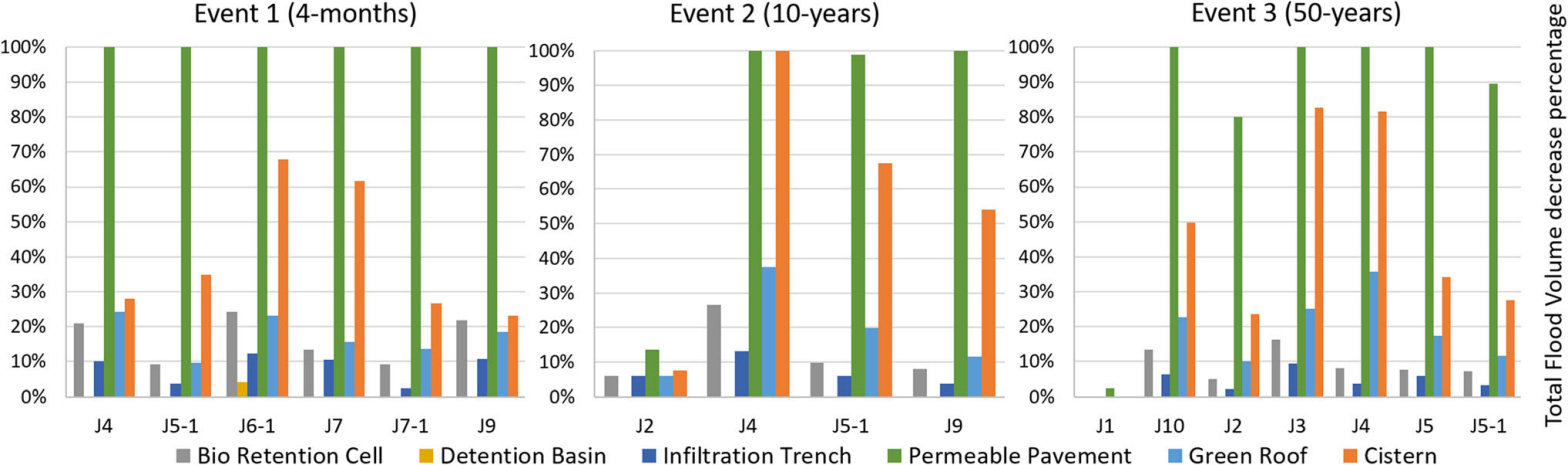
Modelling results for flooded junctions of total flood volume decrease (%) compared to no UGI implementation considering each UGI element alone for the three considered rainfall events. Junctions without an incidence of flooding are not shown

Significant differences in performances are revealed when comparing each UGI element. The highest reductions in flood volume and peak discharge at critical sites (junctions) for all modelled rainfall events result from the implementation of permeable pavement followed by cisterns (see Fig. 5). Compared to the status quo simulation, the full implementation of permeable pavement alone leads to a complete reduction of flood occurrence at all sites for the 4-month simulation and at several sites for the 10 and 50 years return period events. Modelling only cisterns results in a total reduction of flood volume at one site (junction J4) for the 50-year event and reductions of more than 80% for two sites (junctions J3 and J4) for the 10-year event. The scenario with 75% of buildings equipped with green roofs leads to maximum flood volume reductions of close to 40% (junction J4, 10-year event). The assumed maximum implementation of bio-retention cells reaches 26% flood volume reduction at its best performance (junction J10, 10-year event). The implementation of infiltration trenches achieves 13% of flood volume reduction as its best value at the same site and for the same rainfall event. The assumed maximum possible implementation of detention basins does not reduce the flood volume at any critical sites at all while all other UGI elements lead to a reduction of flood volume at all sites for all events except for junction J1 for the 50-year event when only the permeable pavement has a flood reducing effect (see Fig. 5).

**Fig. 6:**
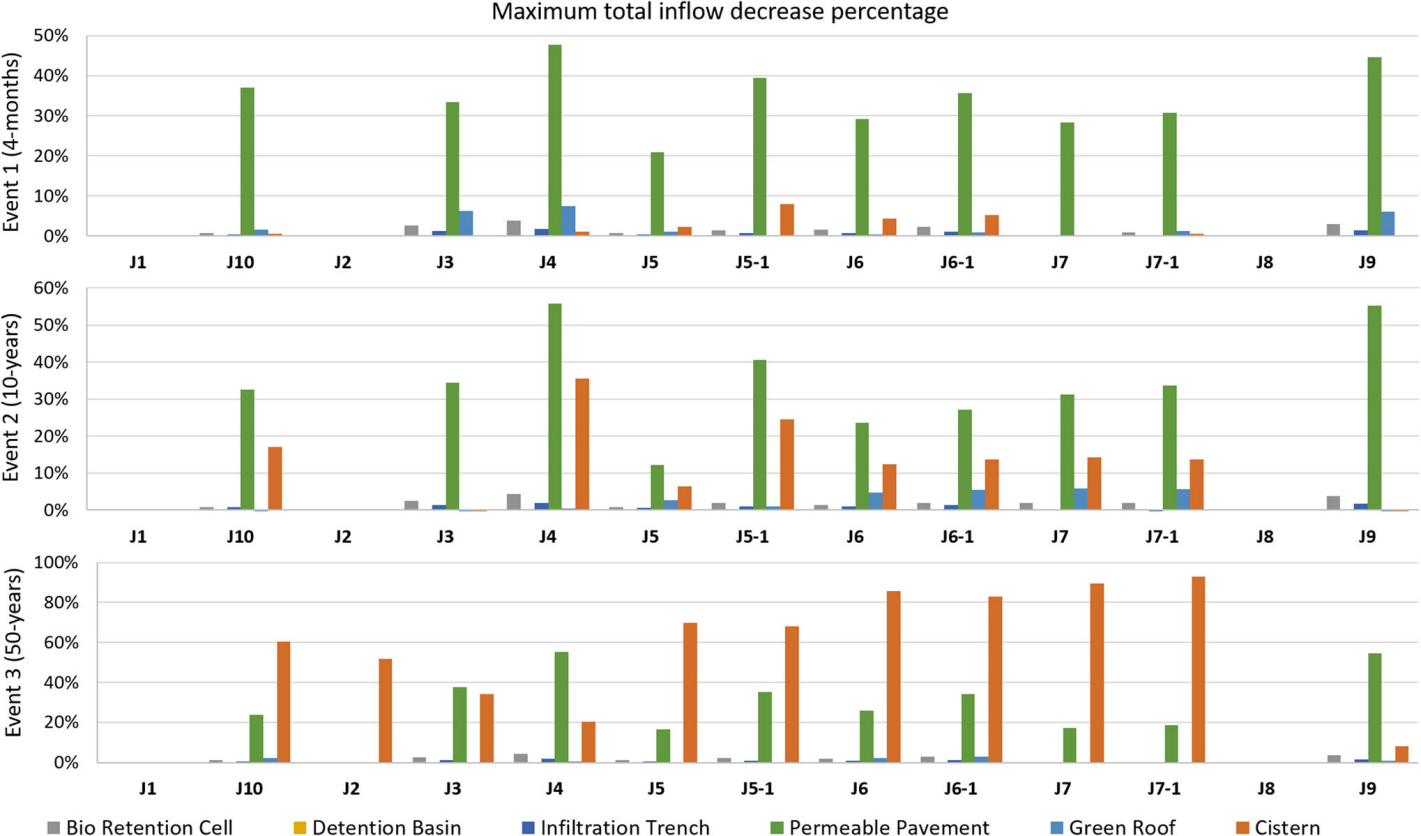
Modelling results of peak runoff decrease (%) compared to no UGI implementation considering each UGI element alone for the three considered rainfall events

Comparing the degree of peak runoff reduction by each UGI option at sites within the catchment, it reveals that cisterns are most effective in peak runoff reduction for the 50-year event (20–93% reduction) and permeable pavement performs best for the 10-year event (16–55%) and the 4-month simulation (20–47%; see Fig. 6). Especially in the 4-months simulation, the permeable pavement significantly out-performs all other options.

## Discussion

### Performance of UGI scenarios and policy implications regarding their implementation

The modelling results regarding the different rainfall events indicate that S1 leads to a higher total runoff volume reduction in all rainfall events and peak runoff reduction compared to S2. S2 is more effective in reducing peak runoff than in reducing total flood volume. Although the area converted to UGI is greater in S2, the storage potential of the UGIs of S2 are much lower than of S1 where additionally infiltration takes place. This explains why S2 is less effective in total runoff volume and peak flow reduction for the intensive rainfall event 3, as the UGI elements soon become saturated and do not have any further runoff reducing function.

The results of peak runoff reduction at different junctions of the catchment yields additional insights regarding an effective placement of UGIs within the catchment as the different UGI elements perform differently depending on the sub-catchment of implementation and its location within the catchment.

The individual performance of UGI elements strongly depends on the available area for their implementation. Respective space availability is based on the in-depth analysis of the representative residential area which resulted in most spatial availability for permeable pavement (17.5% of streets) followed by bio-retention cells (6.5% of streets) and infiltration trenches (3% of streets).

This space availability results from the specific design of streets, sidewalks, green verges, as well as the specific traffic (e.g. percentage of streets with low traffic suitability for permeable pavement) and parking situations (e.g. opportunities to use space of streets to implement bio-retention cells) in the representative neighbourhood. The 0.015% of areas with bare soil or low vegetation representing available unbuilt public space suitable for the implementation of detention basins is another result from the specific distribution of this type of space within the representative neighbourhood. With our more “realistic” modelling UGI scenario (S1) we assumed that this situation and the resulting space availability and suitability for different UGI elements is the same for the rest of the urbanized areas of the catchment considered in this study for UGI implementation.

This linear land cover-based approach is a simplification, and it needs to be validated to what degree this assumption is true or whether the situation (e.g. street hierarchies and design, traffic, available unbuilt public space) differs in other parts of the urban catchment. For instance, in the centre of the District of Heredia (Fig. 1), a major commercial area of the catchment, the distribution of street orders and traffic, as well as the use of space for parking, could differ from the assumed representative residential neighbourhood in this study. The total potential of streets suitable for permeable pavement as well as available space for bio-retention cells could actually be less in other residential areas than in the representative neighbourhood.

In this case, the assumption of a linear relationship between the land cover distribution and the available space for UGI elements could overestimate available space for more urbanized areas than the representative neighbourhood. But it could also underestimate the full UGI implementation potential for less densely urbanized areas. Also the Free Trade Zone in the Southwest of the Canton of Heredia (Fig. 1) could potentially have more suitable space for permeable pavement, bio-retention cells, and infiltration trenches compared to residential areas such as the representative neighbourhood, because large parts of the “street network” are parking lots and low traffic areas. In the catchment considered in this study, there are only a few industrial areas.

It is important to determine how representative an area is when considering its UGI potential. In our study, we assessed representativeness based on similarities in land cover distributions and urbanization as well as road network and traffic intensity characteristics. The larger and more heterogeneous the area that is supposed to be represented becomes, the greater uncertainty about whether this assumption is still valid and the modelling still correct. Thus, the approach is most suitable for a context where the basic land cover and street network distribution is similar across the identified area. For large heterogeneous landscapes, it may be necessary to identify several representative areas which can be assigned to respective reoccurring land cover patterns implying different potentials for UGI. If the traffic intensity is important for the UGI potential of streets, the use of a traffic simulation software could be useful to identify it. But the street network connections beyond the hydrological boundaries of the catchment would have to be taken into account as well.

Nevertheless, this study’s approach is advantageous and viable for relatively homogeneously urbanized small-to-medium-sized catchments and should result in more realistic scenarios than those not considering space availability at all (Chaosakul et al. 2013; Jia et al. 2014; Joksimovic and Alam 2014; Palla and Gnecco 2015). A greater degree of certainty about whether modelled UGI scenarios can realistically and feasibly reduce flooding is practical information for stakeholders and decision-makers. The scenario building approach presented in this study provides this greater certainty.

For S2 (green roofs and cisterns/rain barrels) no detailed insights regarding the suitability of houses and properties from a representative area were taken into account, only the information of typical roof and property sizes. Thus, this scenario is not directly related to suitable available space, but is progress towards the adoption of these UGI elements on private properties.

A different kind of study to assess the feasibility of replacing existing roofs with green roofs from a structural point of view is needed. The current study explored this tentatively. However, it was assumed that a maximum of 25% of rooftops could be converted into green roofs and 75% of all properties could use rain barrels to assess the potential of UGI elements implying a different policy approach for its promotion. Different policy approaches, at least for the promotion of green roofs on private properties, would be necessary. In particular, buildings in industrial areas or of public institutions may be more suitable for green roofs and easier to implement. Nevertheless, the results for green roofs are of high interest to urban planners and urban water managers dealing with the potential benefits of such measures to reduce urban runoff, especially in areas with high urbanization rates such as the metropolitan area in Costa Rica (Oreamuno and Villalobos 2015).

For the retrofitted UGI elements of S1 located within the street network (permeable pavement, infiltration trench, bio-retention cells), a successive implementation when (residential) streets, sidewalks or street crossings are refurbished could be an effective strategy, especially if the alternative UGI street design is not costlier than the traditional street design. Especially larger unbuilt public spaces could be used for the implementation of detention basins at any time. Since the assumed available space for detention basins was limited in the representative residential neighbourhood (only 0.015% of unbuilt public space), detention basins may be an option for less urbanized sub-catchments or integrated in a multi-functional design of playgrounds or parks for temporal rainwater detention. Municipalities and national institutions defining street and public space design standards are key actors for the ongoing promotion and implementation of all UGI elements of S1. Alternative street designs which allow the integration of bio-retention cells, infiltration trenches, as well as promotion and incentives for the use of new construction materials for permeable pavements in suitable streets could lead to a step-wise implementation.

The UGI elements consider in S2 aim at public or private properties as places of implementation. Municipalities could provide incentives (e.g. co-financing or technical assistance) here for the adoption of greener technologies such as green roofs and rain barrels. A collaboration with the housing industry (e.g. by creating a market for rainwater harvesting technologies) and the water supply sector (to use rainwater as an alternative water source to reduce the negative impact of water shortages) could result in productive synergies. However, the results from this study suggest that the measures simulated on private properties are less effective for rainfall events with return periods of more than 10 years.

As the 4-month modelling results without UGI elements for the measured rainfall time series show, the frequent occurrence of flooding within the catchment is already a pressing problem that needs to be resolved as soon as possible. The results suggest an implementation of retrofitting UGI could significantly reduce flooding, however the scenario of a full implementation is probably not achievable within the next two decades. This means additional measures for large scale flood volume reductions along the river course could be necessary. It is recommended that an analysis of potential sites for these measures and a subsequent modelling of their flood reducing impact is conducted. Afterwards, a cost–benefit comparison between large scale measures along the river course and small-scale retrofitted measures within urbanized areas should be carried out to identify optimal combination strategies.

UGI in residential areas can be designed to offer direct additional benefits to residents such as local climate regulation, reduced air pollution and traffic, aesthetic improvements, more public space for recreational activities, and also the treatment of grey water and water reuse (Kim 2018). These direct and on-site benefits can be more persuasive in gaining social acceptance for the promotion and implementation of these measures. Bio-retention cells, detention basins, and green roofs bear the potential to provide additional social and ecological benefits beyond hydrological ones (Ministry of the Environment 2003; Kellagher and Laughlan 2005). In contrast, large scale measures along the river course often have a more complex cost–benefit-sharing constellation with upstream costs and downstream benefits. If cost and benefits occur in different administrative units (e.g. municipalities), cost-sharing mechanisms will probably become necessary.

### Methodology and modelling approach in the context of other studies

A similar approach to the presented study was conducted by Ahiablame and Shakya (2016), who used PCSWMM to model the flood attenuation effects of porous pavement, rain barrels, and rain gardens at various application levels in an 87 km^2^ catchment in Illinois, USA, for a period of 30 years with scenarios of increasing urbanization (50–94%). The three considered UGI practices lead in their study to between a 3 and 47% runoff reduction and between a 0 and 40% reduction of flood flows, indicating that UGI practices can be used to mitigate flood risk in urban catchments. However, their study focused on probable urbanization scenarios and not on realistic retrofitting UGI scenarios. The study identifies permeable pavement to be the most effective UGI to reduce runoff in the Sugar Creek Watershed, Illinois. But scenarios ranging from 25 to 100% of street conversion to permeable pavement, whereas in this study a maximum of 17.5% of street conversion was seen as realistic. Although the effectiveness of UGI elements to reduce runoff and flood volume was shown, the question of whether the simulated UGI elements could be realistically implemented was not addressed.

Vittorio and Ahiablame (2015) used PCSWMM to investigate the hydrologic effects of upscaling UGI practices (rain barrels, rain gardens and porous pavement) to the catchment scale in a 93 km^2^ highly urbanized catchment of Missouri, USA. This study showed that UGI practices could be used to restore pre-development hydrologic conditions by achieving runoff reductions from 3 to 31% with increased UGI implementation levels (from 25 to 100% for each of the UGI options).

Both studies employed a linear incremental UGI scaling approach with 25–100% degree of implementation. These studies provide hypothetical results about the relative performance of different UGI strategies. However, in contrast to this study’s results, they do not provide information regarding the potential of UGI to be realistically implemented considering spatial constraints.

Few studies of UGI implementation in Latin American countries exist. A recent study Jiménez Ariza et al. (2019) conducted in Bogota, Colombia, provided a methodological proposal for the selection and placement of UGI elements in consolidated urban areas. The study resulted in the identification of priority implementation areas, strategic plans, and most suitable UGI elements for public areas in Bogota, but the impact of runoff reduction of UGI scenarios were not modelled or quantified. Nevertheless, such research studies can be helpful to guide hydrological modelling studies, e.g. in the development of UGI implementation scenarios to be modelled.

## Conclusions

Despite the broad recognition of the benefits of UGI, its implementation has largely remained limited to rather small areas. Hence, the potential impacts of flood reduction due to large scale implementation of UGI can only be investigated with the use of models. With the development of UGI scenarios based on detailed information regarding implementation constraints from an in-depth representative neighbourhood study, this study provides a more reliable and accurate estimation of how a catchment-wide implementation of UGI in urban areas with similar characteristics could reduce flooding. With the proposed methodology, both the individual and combined effect of different UGI elements for different precipitation events can be assessed. The comparison of an UGI scenario limited to public space and an UGI scenario limited to properties reveals the potential of two different implementation strategies.

In comparison to previous UGI implementation studies, this study accounts for specific site constraints in retrofitting contexts of public space. These site-specific constraints reflect the urban neighborhood development characteristics of Latin American cities. Future studies that account for spatial constraints typologies of different neighbourhoods in the development of UGI upscaling scenarios are recommended. This approach would be of particular value for urban areas in the Global South, where UGI implementation studies and strategies are still lacking. Given that a large share of present and future urban areas are and will be located in tropical countries with informal urbanization characteristic and the respective specific implementation constraints, there is a need to further study possibilities of UGI implementation and to develop realistic implementation strategies. The results presented here may guide policy making to promote future UGI implementation strategies to reduce flooding.

## Supplementary Information

Below is the link to the electronic supplementary material.

(PDF 3195 kb)
