# Correction to: Development and modelling of realistic retrofitted Nature-based Solution scenarios to reduce flood occurrence at the catchment scale

**DOI:** 10.1007/s13280-021-01535-9

**Published:** 2021-03-05

**Authors:** Valerie Chen, Jose Ricardo Bonilla Brenes, Fernando Chapa, Jochen Hack

**Affiliations:** 1 Department of Civil and Environmental Engineering, Technical University Darmstadt, Franziska-Braun-Str. 7, 64287 Darmstadt, Germany; 2 Research Group SEE-URBAN-WATER, Section of Ecological Engineering, Institute of Applied Geosciences, Technical University Darmstadt, Schnittspahnstr. 9, 64287 Darmstadt, Germany

## Correction to: Ambio 10.1007/s13280-020-01493-8

In the original publication, the affiliation of all the authors were mixed up and processed incorrectly. It has been updated in this correction.

The original article has been corrected.

