# Supplementary material for: Development and modelling of realistic retrofitted Nature-based Solution scenarios to reduce flood occurrence at the catchment scale
Source: Ambio. 2021 Jan 26;50(8):1462–76. doi: 10.1007/s13280-020-01493-8 (PMC8249552; doi:10.1007/s13280-020-01493-8)
Supplement: Supplementary file 1 — (PDF 3195 kb) [file 13280_2020_1493_MOESM1_ESM.pdf]

# Ambio

Electronic Supplementary Material

*This supplementary material has not been peer reviewed.*

Title:

**Development and Modelling of realistic retrofitted Nature-based Solution Scenarios to reduce Flood Occurrence at the Catchment Scale**

Authors:

Valerie Chen, Ricardo Bonilla Brenes, Fernando Chapa and Jochen Hack

# 1 Supplementary material

## Appendix S1. Characteristics of the representative area and placement potential for each UGI.

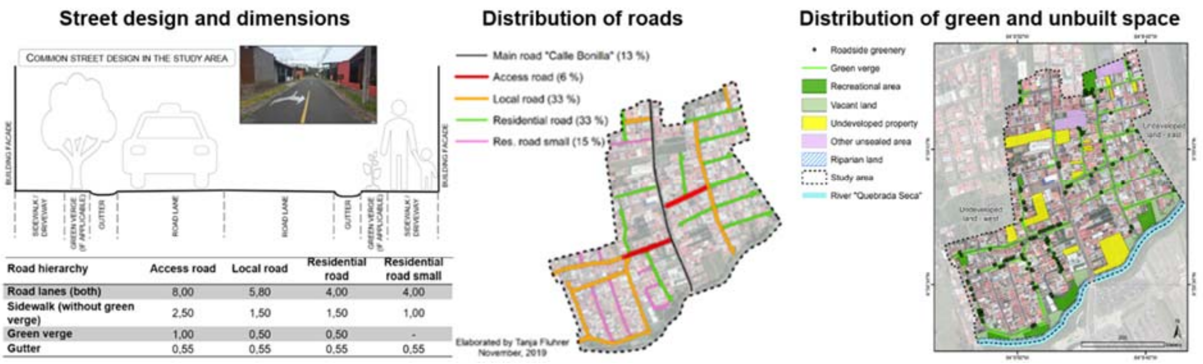


Fig. S1: Landscape characteristics that were considered as constraints to a realistic implementation of UGI elements: (Left) street design and dimensions of different hierarchical roads, (middle) spatial distribution of road hierarchy, and (right) existing green network and available open space for UGI in the representative neighbourhood (Fluhrer and Hack 2020).

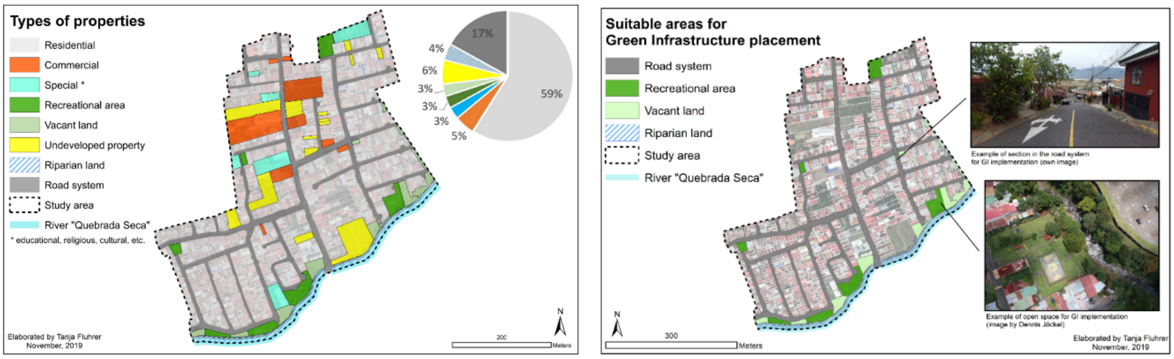


Fig. S2: Land-use and areas considered as suitable for the placement of UGI within the representative neighbourhood (Fluhrer and Hack 2020).

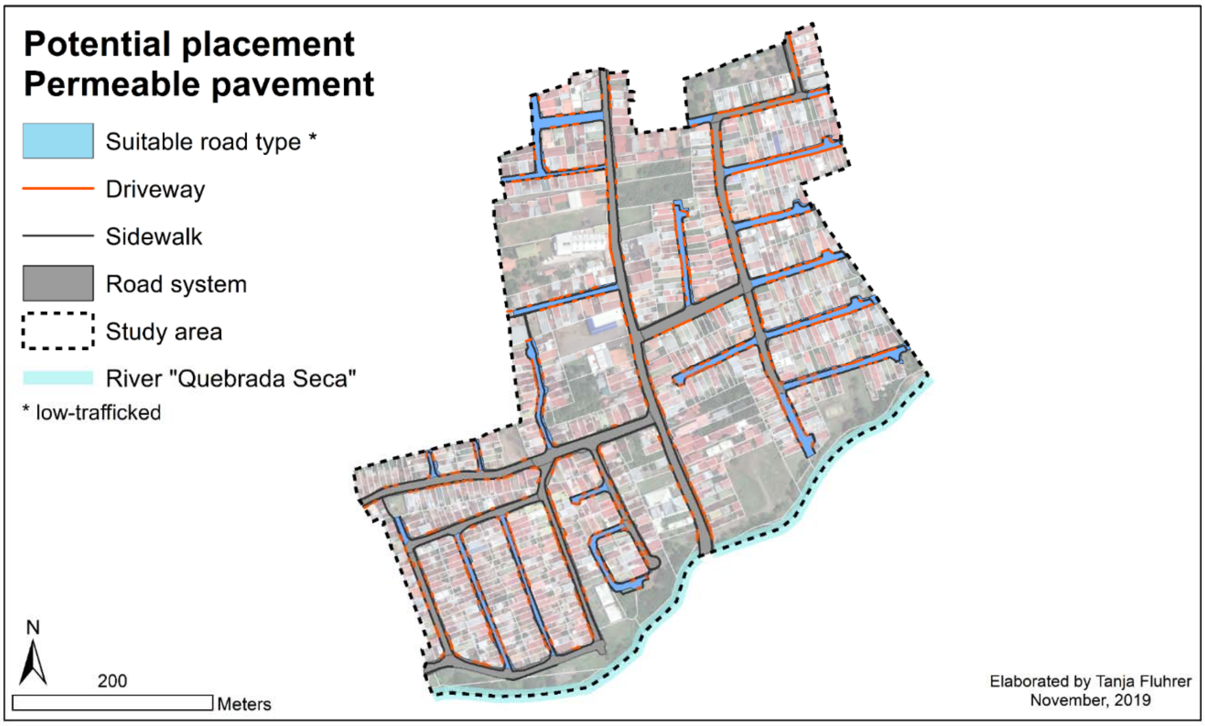


Fig. S3: Potential placements for permeable pavement identified in the representative neighbourhood (Fluhrer and Hack 2020). Source of background image: Google Earth.

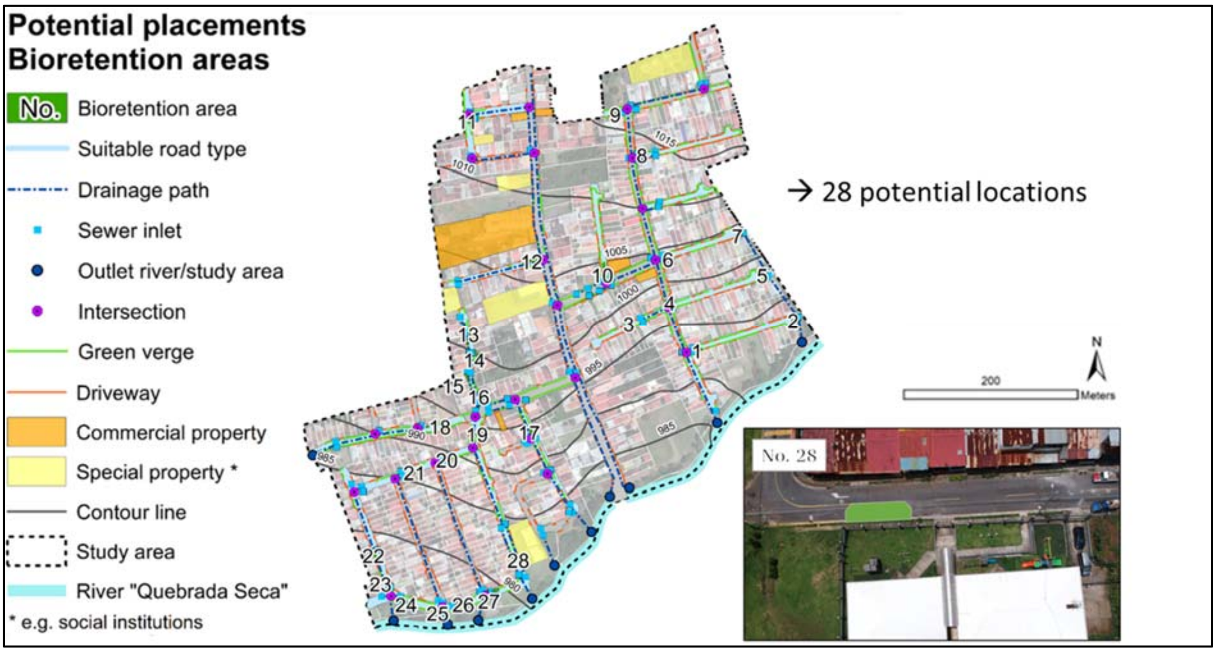


Fig. S4: Potential placements for bio-retention areas identified in the representative neighbourhood (Fluhrer and Hack 2020). Source of background image: Google Earth.

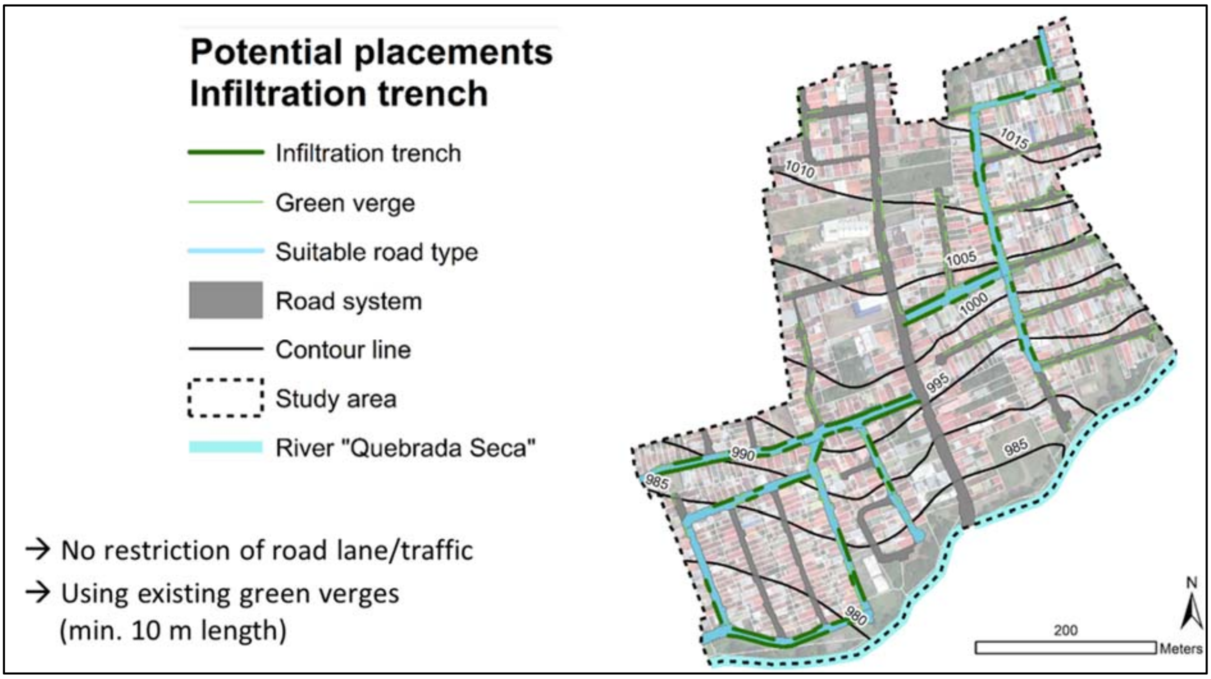


Fig. S5: Potential placements for infiltration trenches identified in the representative neighbourhood (Fluhrer and Hack 2020). Source of background image: Google Earth.

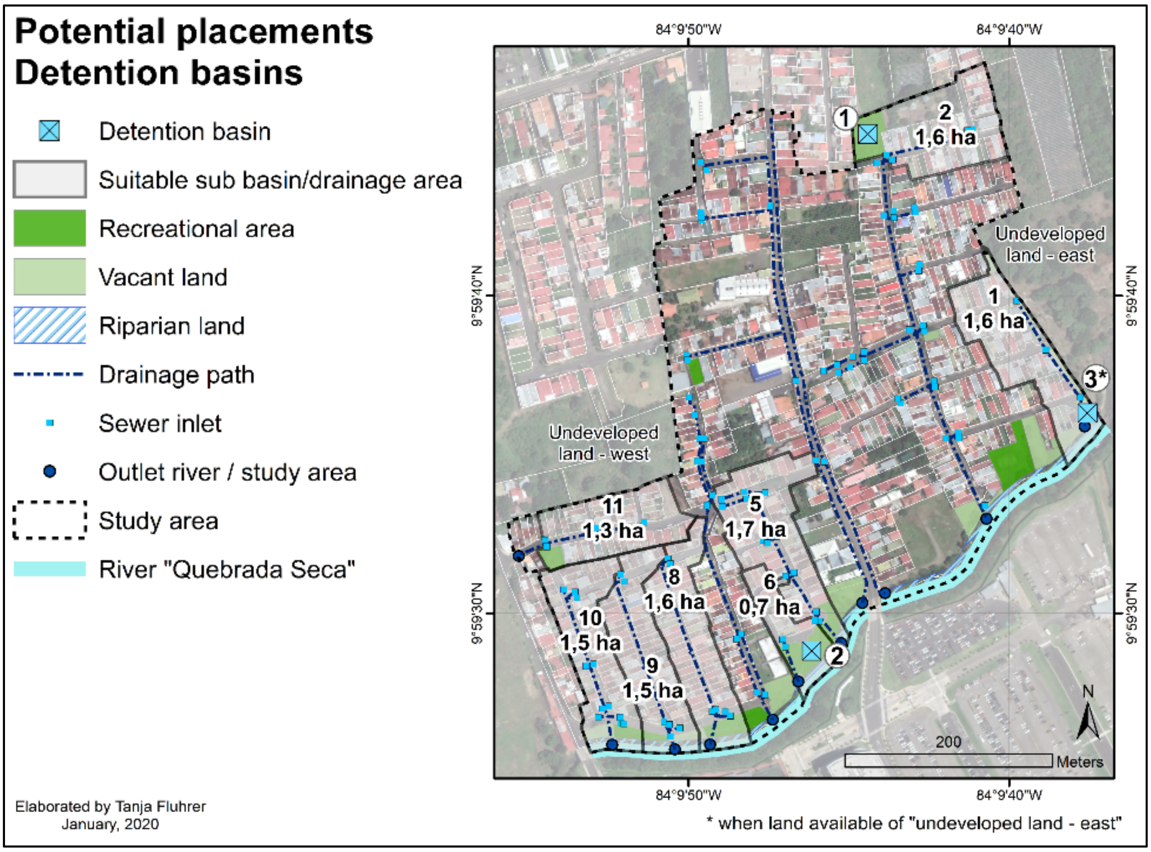


Fig. S6: Potential placements for detention basins identified in the representative neighbourhood (Fluhrer and Hack 2020). Source of background image: Google Earth.

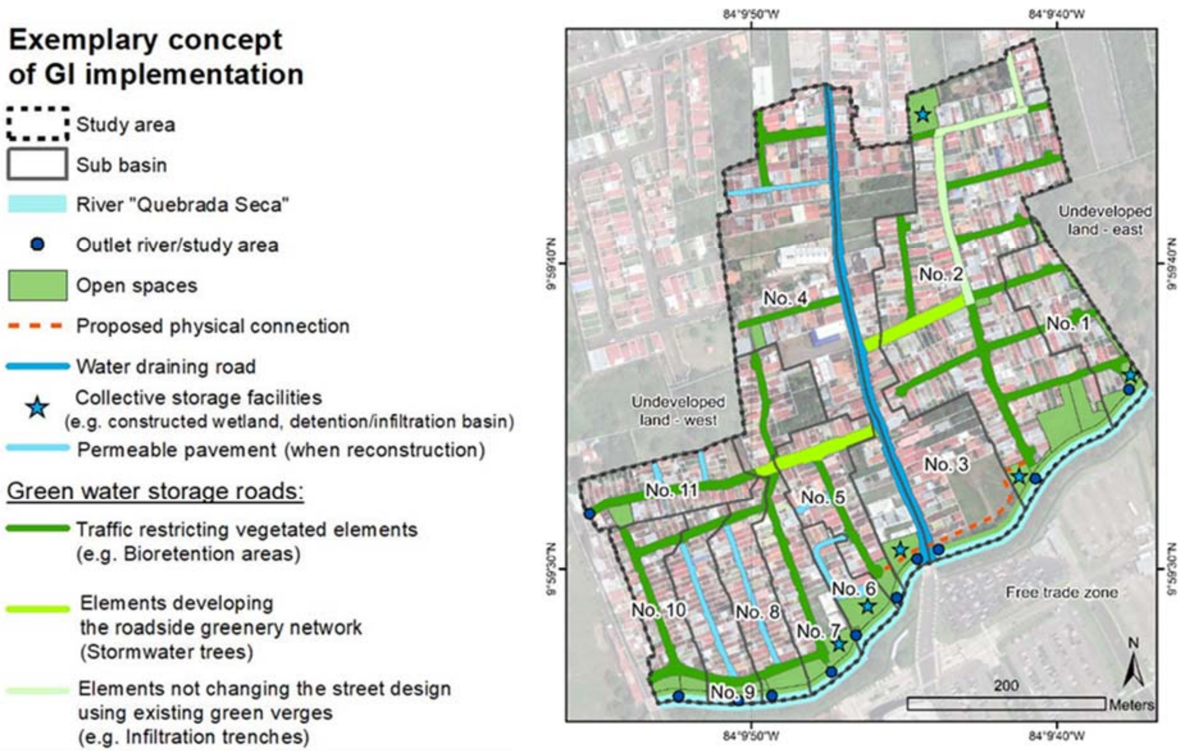


Fig. S7: Maximum realistic potential for Urban Green Infrastructure implementation in public space within the representative neighbourhood (Fluhrer and Hack 2020).

**AppendixS2. Characteristics and model parametrization for each subcatchment**

Table S1: Area and land cover characteristics of the representative area and the sub-catchments employed in PCSWMM to model the Quebrada Seca catchment. Sub-catchments highlighted in grey are considered as critical due to their high degree of impervious area and relative share of contribution to flooding

| Sub-catchment | Total Area [ha] | Area bare soil | Area high vegetation | Area low vegetation | Impervious Area |
|---|---|---|---|---|---|
| A9 | 235.6 | 27% | 9% | 14% | 49% |
| A8 | 228.2 | 31% | 23% | 27% | 18% |
| A7-2 | 30.0 | 37% | 12% | 28% | 23% |
| A7-1 | 68.4 | 28% | 16% | 14% | 42% |
| A7 | 139.6 | 33% | 18% | 20% | 29% |
| A6-1 | 233.3 | 29% | 4% | 10% | 58% |
| A6 | 173.7 | 25% | 5% | 15% | 55% |
| A5-1 | 197.1 | 27% | 8% | 13% | 52% |
| A5 | 28.3 | 29% | 7% | 18% | 46% |

| | | | | | |
|---|---|---|---|---|---|
| A4-1 | 67.7 | 22% | 17% | 19% | 42% |
| A4 | 195.5 | 22% | 9% | 11% | 58% |
| A3 | 117.1 | 27% | 9% | 15% | 48% |
| A2 | 73.7 | 32% | 18% | 22% | 28% |
| A11 | 100.7 | 26% | 7% | 13% | 55% |
| A10 | 126.2 | 28% | 14% | 21% | 37% |
| A1 | 231.8 | 26% | 29% | 34% | 10% |
| A0 | 38.5 | 25% | 30% | 33% | 12% |
| Representative area | 33.0 | 20% | 7% | 14% | 59 % |

Table S2: Overview of the model input data with data type, resolution, date of origin / period of time, source and processing of the used data

| **Data type** | **Resolution** | **Source** | **Date / Period** | **Processing** |
|---|---|---|---|---|
| Land use | 0.5 m Pixel resolution | Satellite image from Google Earth Pro | January 2019 | Land use classification in QGIS with “Semi-Automatic Classification Plugin” |
| Soil data | n.a. | Ministry of Agriculture and Livestock | 1991 | n.a. |
| Potential evaporation | Monthly mean values | UN data; Station Juan Santamaría | Values based on the period 1971 – 1990 | Conversion to actual evaporation with the factor 0.7 |
| Precipitation | 5 min | Municipality of Belén, and University of Costa Rica – CIEDES | Since July 2017 | n.a. |
| Runoff | 5 min | Hydrostatic pressure sensor (TD-Diver™ and Baro-Diver®) in Flores | Since June 2019 | Hydrostatic pressure equation and Gauckler-Manning Formula to calculate runoff |

Table S3: Model parametrization for each sub-catchment. Parameterization of Drying Time and Curve Number based on (USDA 1986; Oreamuno Vega and Villalobos Herrera 2015; Rossman and Huber 2016)

| **Name** | **Area (ha)** | **Width (m)** | **Flow Length (m)** | **Slope (%)** | **Impervious (%)** | **Drying Time (days)** | **Curve Number** | **Soil type** |
|---|---|---|---|---|---|---|---|---|
| A0 | 38.5 | 573.0 | 671.9 | 10.5 | 12.3 | 5.0 | 24.7 | Zarcero, Concepción |
| A1 | 232.0 | 1163.2 | 1994.3 | 12.7 | 10.5 | 8.4 | 30.5 | Zarcero, Concepción |
| A10 | 126.3 | 945.5 | 1335.6 | 6.3 | 37.0 | 4.3 | 34.9 | Heredia |
| A11 | 100.8 | 819.4 | 1229.9 | 7.9 | 54.7 | 4.3 | 36.8 | Heredia |

| | | | | | | | | |
|---|---|---|---|---|---|---|---|---|
| A2 | 73.7 | 546.3 | 1349.5 | 12.8 | 28.2 | 9.1 | 34.9 | Concepción, Heredia |
| A3 | 117.2 | 1069.6 | 1096.0 | 9.2 | 48.2 | 5.0 | 36.1 | Concepción, Heredia |
| A4 | 195.7 | 1390.5 | 1407.4 | 7.4 | 57.8 | 4.3 | 35.9 | Heredia |
| A4-1 | 67.7 | 576.8 | 1174.0 | 8.1 | 42.4 | 4.3 | 33.8 | Heredia |
| A5 | 28.3 | 441.2 | 641.0 | 6.0 | 45.5 | 4.3 | 36.3 | Heredia |
| A5-1 | 197.2 | 1400.0 | 1408.8 | 5.7 | 52.3 | 4.3 | 36.6 | Heredia |
| A6 | 173.9 | 1331.3 | 1305.9 | 6.5 | 55.3 | 4.0 | 36.6 | Alajuela |
| A6-1 | 233.5 | 2187.0 | 1067.5 | 5.9 | 57.8 | 3.7 | 38.3 | Alajuela |
| A7 | 139.7 | 1260.6 | 1108.4 | 7.3 | 28.7 | 4.3 | 35.2 | Alajuela |
| A7-1 | 68.4 | 458.4 | 1493.0 | 6.2 | 42.2 | 4.3 | 35.3 | Alajuela |
| A7-2 | 30.0 | 410.7 | 731.0 | 6.2 | 23.2 | 4.3 | 35.4 | Alajuela |
| A8 | 228.4 | 848.0 | 2692.9 | 11.2 | 18.1 | 10.9 | 33.9 | Zarcero, Concepción |
| A9 | 235.8 | 1176.6 | 2004.2 | 7.3 | 49.1 | 4.5 | 36.1 | Concepción, Heredia |

Table S4: Soil type parametrization (Oreamuno Vega and Villalobos Herrera 2015)

| **Name** | **Zarcero** | **Alajuela** | **Concepción** | **Heredia** |
|---|---|---|---|---|
| Soil type | I 099 | I 108 | I 110 | I 111 |
| Texture | loam (medium) | loam/clay-loam | clay, fine | loam/clay-loam |
| Slope | 12-25% | 10-20% | 10-20% | 12-25% |
| Matrix material | Volcanic ashes | Ignimbrites with volcanic ashes mantles | Volcanic ashes and lava | Volcanic ashes over lava and lahars |
| Drainage | Good | Moderate - Good | Good | Good |
| Water level (cm) | +120 | Deep | Deep | +120 |
| Average annual precipitation (mm) | 2550-5680 | 1900-2400 | 2300-2800 | 1900 |
| Average annual temperature (°C) | 15-18 | 21-26 | 18-24 | 21-24 |
| Pedological horizons | | | | |
| Ap | Sandy loam | Loam | Clay | Clay |
| A2 | Loam | Loam | | |
| AB | ND | Clay loam | | |
| Bw1 | Sandy loam | Clay loam | | |
| Bw2 | ND | Clay loam | | |
| BC | Loam | Clay silt | | |
| Max depth (cm) | 150 | 113+ | 85 | 145 |
| Hydrologic soil group | A | B | B | B |

**Appendix S3. Calibration and validation**

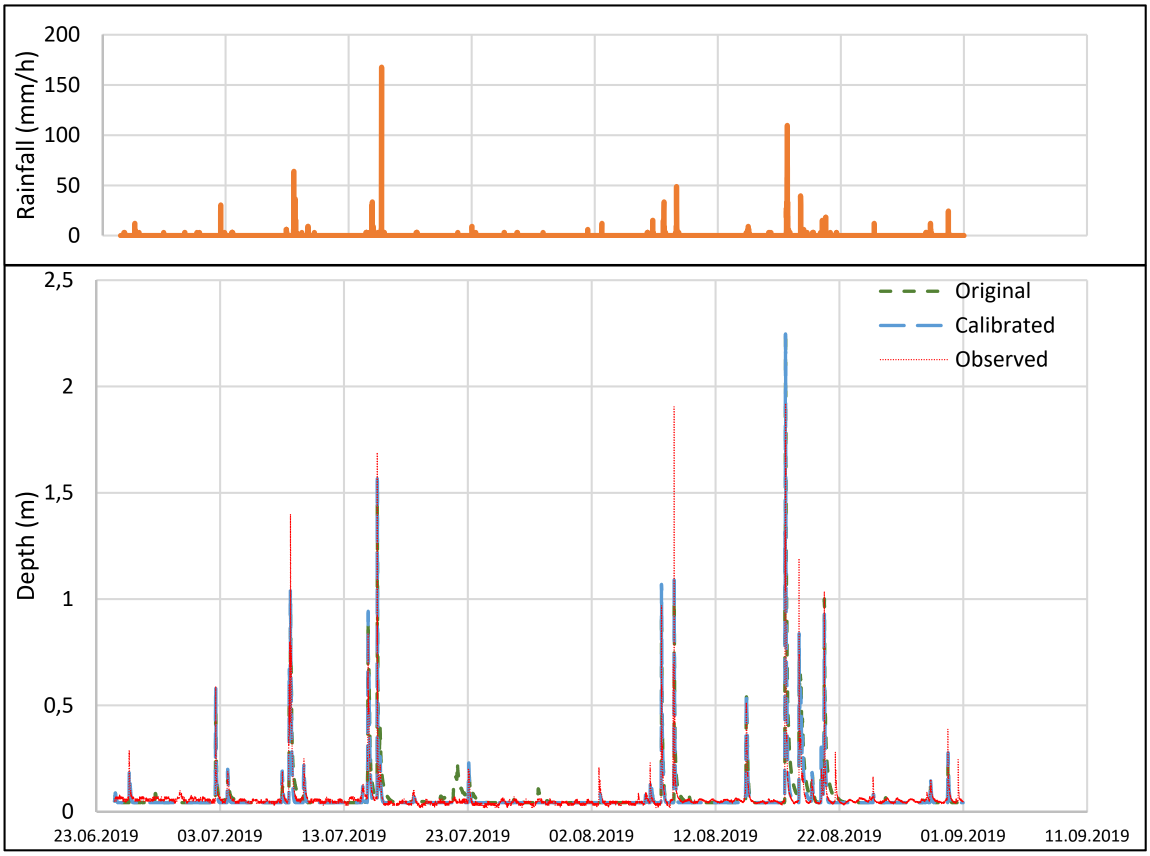


Fig. S8: Control point depth data and rainfall distribution after sensitivity analysis and calibration

Table S5: Error coefficients for calibrated model

| Error | Original | Calibrated |
|---|---|---|
| Integral square error rating | Excellent | Excellent |
| Integral square error (ISE) | 0.607 | 0.424 |
| Nash-Sutcliffe efficiency (NSE) | 0.503 | 0.758 |
| Coefficient of determination (R2) | 0.636 | 0.774 |
| Standard error of estimate (SEE) | 0.08 | 0.0559 |

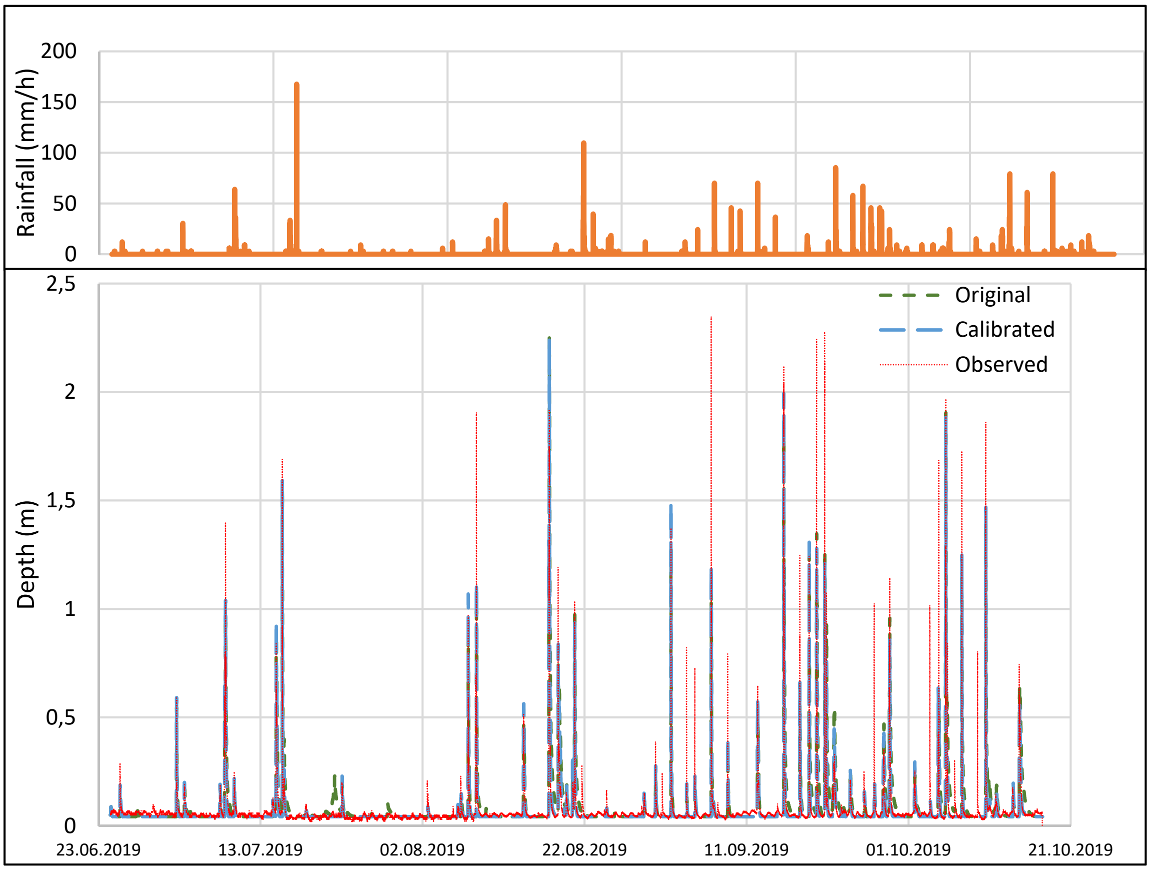


Fig. S9: Control point depth data and rainfall distribution for complete period
